# Analysis of subjective well-being in European Union countries: Group DEA and NPE

**DOI:** 10.3389/fpubh.2025.1570113

**Published:** 2025-07-10

**Authors:** Marta Dziechciarz, Maciej Szczeciński

**Affiliations:** ^1^Department of Econometrics and Operations Research, Wroclaw University of Economics and Business, Wroclaw, Poland; ^2^Department of Mathematics and Cybernetics, Wrocław University of Economics and Business, Wrocław, Poland

**Keywords:** overall life satisfaction, subjective well-being, Ward hierarchical clustering, group data envelopment analysis, group DEA; group non-profit efficiency, group NPE

## Abstract

**Introduction:**

The research aims to analyse the well-being in European Union countries’ identified groups and indicate countries with efficient socio-economic policies. The study explores the differences in households’ subjective well-being in the EU in 2022. The research is a two-pronged examination of selected factors determining well-being in the identified homogeneous groups of EU countries.

**Methods:**

The clustering approach focused on similarities in “inputs and outputs” variables, incorporating key determinants of public health, particularly healthcare system efficiency, social protection policies, and education quality. The evidence-based approaches to health equity and public health assessment include grouping procedures based on variables, which may be defined as “inputs” in social policy and clustering based on variables describing well-being as outcomes in social policy. Furthermore, the efficiency of selected social policy areas in homogenous groups of EU countries was evaluated. The twofold efficiency analysis included Data Envelopment Analysis (DEA) to conduct a deepened study on efficient countries and the Non-Profit Efficiency (NPE) method, both supporting social policy recommendations design aimed at enhancing better health policy outcomes and health-related well-being. It is advisable to compare efficiencies among groups of homogeneous countries. The country’s benchmark should be the DMU, which has similar socio-economic characteristics and health system features. Thus, the classification approach is justified and enables the drawing of much more reliable guidance and the fostering of social policies that contribute to better public health outcomes.

**Results and discussion:**

The originality lies in establishing efficiency benchmarks in a two-step analysis involving clustering and efficiency determination with a direct application to developing sustainable social policies. The results of the modified NPE analysis were juxtaposed with benchmarks and targets obtained in DEA, additionally supporting recommendations for improving well-being and social policy effectiveness in the EU.

## 1 Introduction

Discussion about the efficiency of socio-economic policy is of great importance these days. The subjectively perceived well-being of societies and their parts is the subject of economic and social policy interest. The residents’ sense of well-being is an essential indicator of a country’s development and a critical social issue that significantly affects perception of citizens’ own situation (53). Raising the level of perceived well-being is considered a measure of the effectiveness of the socio-economic policy of the governments of individual countries (54). This study emphasises the role of health policy aimed in inequalities reduction and healthcare services quality. Our research is in line with the guidelines set out in the UN’s Sustainable Development Goals, Good Health and Well-Being (Goal 3).

Nowadays, there is an in-depth discussion in the literature on defining the terms concerned with households’ subjective well-being perception. The comprehensive debate on the quality of life and the material well-being of citizens and families is at the outset (55, 56). Since there has been a considerable increase in material well-being levels, the issue of subjective well-being has gained in the social sciences. Parallel, new developments in the positive psychology and health research heightened deepened interest in well-being (1, 57–60).

The purpose of the research is to analyse the well-being of European Union countries through a deepened study into subsamples (groups of homogenous European Countries). The hypothesis was that public expenditures were efficient in those countries with elevated indications of the subjective well-being of households (54, 61, 62). A key assumption is that social policies play a crucial role in shaping well-being outcomes. Our hypothesis stated that analysis in subsamples (groups of homogenous European Countries) would enable the discovery of partial associations or partial patterns. The whole sample’s analysis may sometimes lead to latent association omissions. This reasoning is consistent with the Simpsons’ paradox. Due to paradox, aggregated data may show relationships that are not present (or will be reversed) in subpopulations of the data.

The research follows a two-pronged approach, examining selected factors of well-being in the identified homogenous groups of European Union member countries. Data Envelopment Analysis (DEA) and Non-Profit Efficiency (NPE) methods were used to evaluate the effectiveness of Decision Making Units (DMUs). A key assumption for the analytical technique (DEA) is that DMUs are homogenous. In practice, this assumption means that DMUs should have the same technology. In the case of analysing the effectiveness of social policy aimed at generating subjective well-being of European Countries, the assumption of in DEA is the homogeneity is not fulfilled. Hence, the effectiveness study was conducted on homogeneous subgroups of DMUs, meaning they should share similar socio-economic and technological characteristics. Health and social care policies differ across EU countries, impacting subjective well-being and life satisfaction. To address lack of homogeneity, efficiency studies are conducted within identified subgroups.

The clustering approach focused on similarities in “inputs and outputs” variables related to public health and social care. For this purpose, two classification tasks were formulated. One grouping is based on variables, which may be defined as “inputs” in social policy. For the second classification, we used variables that describe well-being and are “outcomes” in social policy. The classification approach enables the drawing of much more reliable recommendations. It avoids evaluating heterogenic countries and indicating their benchmarks from different socio-economic spaces, thus ensures more reliable policy recommendations. Furthermore, the efficiency of selected social policy areas in the homogenous groups of EU countries (between groups) was evaluated. The twofold analysis included DEA covering a deepened study on efficient countries and Non-Profit Efficiency (NPE) in all clusters and both classifications. By avoiding comparisons across vastly different socio-economic contexts, the study ensures more reliable policy recommendations.

The studied set of EU member countries is heterogenic. Grouping the sample countries was to discover whether a reversal paradox, such as Simpson’s paradox, is present in the data or if only a change of magnitude in the associations occurs (63, 64). Furthermore, analysing the pattern changes in the sample and sub-samples could lead to pointing out the control variable (or control variables).

Two multivariate classifications cover approach based on “input variables” related to social policy (e.g., healthcare spending and social care support). The second one based on “output variables” describing subjective well-being (e.g., self-reported life satisfaction and perceived well-being). The clustering technique used for the purpose was a method known as hierarchical clustering. Input-based classification leads to defining homogenous groups of European countries in terms of expenditures on social policy. Output-based classification leads to finding homogenous groups of European countries regarding subjective well-being levels (2, 3).

Afterwards, a DEA output-oriented study was done for countries that are similar in terms of “Input variables.” DEA output-oriented ought to show which countries are the most efficient with analogous inputs. The next step of DEA covers a deepened study of efficient countries from all clusters. In the obtained groups based on “Output variables,” DEA input-oriented was conducted. The group of countries are homogeneous in terms of “Output variables”; thus, DEA input-oriented should identify which countries are the most efficient with similar outputs. The second step of DEA covers a deepened study on efficient DMUs from all clusters in both classifications. Supplementary analysis, applying NPE leads to further findings that the DEA method could not clarify sufficiently into effectiveness of health and social care policies in improving well-being.

Additional motivation to investigate efficiencies in obtained groups of European Countries is socio-economic discrepancies between countries. Efficiency comparisons are more methodologically sound when conducted among groups of countries that are similar in terms of “input” or “output” variables.

Socio-economic changes including health system improvements and social policy reforms require time and sustained investment, thus take much time and effort. It is a long and complicated process. Therefore, the first counsel should not be that the country’s benchmark is the DMU from different socio-economic spaces. The classification approach is justified and enables the drawing of much more reliable recommendations.

The originality of our concept in this study lies in establishing efficiency benchmarks for social and public health policies through a two-step analysis process of clustering and efficiency determination. The NPE method was modified and adapted to fit the specificity of the research. Additionally, the results of the NPE, including benchmarks and targets, analysis were compared with benchmarks obtained in DEA.

The importance of the approach lies in identifying the types and directions of most effective expenditure on the sustainable social policies that enhance public health that generate the most significant increases in the subjective perception of well-being. The DEA (and NPE) method was preceded by a classification approach to distinguish homogeneous subgroups among EU members. Identifying the most effective socio-economic policy tools can be seen as a tool for optimising the types and directions of expenditures, leading to maximising effects at given expenditure levels.

## 2 Theoretical framework

It is crucial to recognise that efficient allocation of public resources directly influences the determinants of SWB, such as healthcare quality, education access, and income support. It is the way to bridge public spending efficiency and subjective well-being. The models described in the literature of the topic highlight that the volume and the efficiency of public spending are crucial to enhance life satisfaction and reduce well-being inequality (4, 5).

Two approaches are common in analysing the efficiency of public spending on subjectively perceived well-being. The sectoral approach consists of assessing the effectiveness of the expenditures in separate sectors, i.e., education, health care, and social care. By integrating different public policy sectors into a coherent whole, the holistic (global) approach is gaining increasing importance. The all-inclusive approach acknowledges interdependencies and intersectional connections, which allows for better identification of sources of synergies and complementarity effects (6, 7). Notably that the holistic approach considers cross-sectoral synergies and effects of substitution and complementarity and thus includes and appreciates the coherence of public policies.

The importance of the research conducted should be considered on two levels—cognitive and methodological. For socio-economic policy, one of the critical tasks is to improve the subjective perception of well-being (8). With permanently tight national budgets, the strategic task is to achieve the most significant possible effects while optimising resource allocation, particularly in public health, and access to healthcare (9, 10). Not all methods of spending funds on social policy generate effects on the desired level, i.e., they are not equally effective (11, 12).

In response to this demand from politicians, research has been directed towards conceptual, theoretical and empirical aspects of well-being. The conceptual system of defining individual elements of well-being is focused on an individualistic approach (13–15). The level of well-being in territorial-political units, primarily for individual countries and their groups, is determined through the measured level of personal feelings, most often at the level of households or, less frequently, at the level of individuals (1, 16).

Importantly although the term well-being is attached to the individual’s specific person, the household is the research unit in the measurement practice. Therefore, the measurement results depend to a great extent on the statements made by the head of the household (17). As a result, the term subjective well-being perception is alternatively understood as stated by an individual or describes the household’s situation.

To expand and clarify the conceptual distinctions between four commonly referenced constructs in well-being research, i.e., objective well-being, subjective well-being, economic wealth (material well-being), and quality of life, it is essential to emphasise that these terms are not interchangeable. Each represents a distinct analytical dimension of human welfare, with its precise and distinct definition, measurement logic, and relevance for evaluation and policy design.

Objective well-being refers to measurable life conditions, such as income, health status, educational attainment, and living environment. It serves as a baseline for assessing citizens’ social and material situation. Self-reported evaluations of life, including both emotional states (hedonic well-being) and a sense of purpose or meaning (eudaimonic well-being), describe subjective well-being and play a central role in understanding perceived life assessment and level of personal fulfilment. Economic wealth denotes the material and financial resources held by individuals, households, or nations and represents a fundamental determinant of well-being, often used in economic analyses through indicators like income or GDP *per capita*. Quality of life is an integrative concept that synthesises objective living conditions and subjective life evaluations into a multidimensional framework used to assess overall societal well-being.

The key references for formulated clarification, which improve the conceptual structure of the key terms’ understanding, align with the broader well-being literature (18, 19). It also strengthens the interpretability of our subsequent analysis by ensuring that each construct is used with clear boundaries and purpose.

The theoretical framework of well-being at the most general level includes the concept of the dignity of life (20, 21). The idea of the dignity of life combines the quality of life, i.e., an objectively measurable approach, and the subjective perception of well-being, which is based on an approach where individual elements cannot be objectively measured (22). The formulated opinions are based on the subjective feelings of citizens. Subjective well-being assessments, although based on personal feelings, reflect broader social factors of the quality of life, such as healthcare quality and access to essential services (23, 24).

The most general theoretical approach based on measurable indicators used in the description and measurement of social well-being is called the level of quality of life. Quality of life is an approach that includes individual elements relating to people or households (families) and general elements common to more extensive parts of society (1, 14, 16). These general elements include housing, health services, safety, education, quality of democracy, transport and telecommunications infrastructure, the natural environment and its quality, conditions for recreation and other aspects affecting the quality of life, etc.

An equally important element of the measurable approach is quantifying the individual level of economic wealth, the state of possession of financial resources and durable goods. Material elements are usually identified with the economic well-being concept, also called objective economic status or well-being. As with any measurement, the level of well-being can be interpreted only based on reference points (point system). The reference point system introduces an element of subjectivity into the assessment and interpretation. Nevertheless, the results of measuring the individual level of economic wealth may be referred to as the assessment of the objective socio-economic status.

Contrary to the term containing the descriptor “objective,” in the sense of “measurable,” individuals or households assess their condition subjectively, mainly in comparison to the social and sociable environment, i.e., other people or other families. An assessment formulated based on objective, measurable aspects and comparing these values to the values in the immediate environment, in the circle of friends, family members, community leaders, etc., leads to the formulation of an assessment, which in the literature is called indicators of subjective socio-economic status. Starting with the seminal publication of R. Easterlin, researchers have been attaching increasing importance to the subjective perception of well-being (25). Measuring the perception of subjective well-being has entered the arsenal of tools of the most significant statistical institutions, including national statistical offices, Eurostat—the EU-SILC database, OECD, the World Bank and other international organisations. A network of research organisations has been established to collect measurement results. One of the widely used data sources on the subjective perception of well-being (65) is the research organisation OurWorldInData.org.

To understand the measure known as the Cantril Ladder, its development, validation, and comparative context, it is essential to note that the Self-Anchoring Cantril Ladder, introduced by Hadley Cantril in 1965, is now a widely used tool for assessing subjective evaluative well-being. Data collection involves asking respondents to imagine a ladder with levels numbered from 0 (representing the worst possible life) to 10 (the best possible life) and to indicate the level that best reflects their current life situation. This single-item measure has gained importance due to its simplicity and applicability across cultural contexts. It is a staple in large-scale surveys such as the Gallup World Poll and the World Happiness Report.

The Cantril Ladder has been the subject of numerous validation studies. An example of a recent study is of young refugees experiencing post-traumatic stress symptoms (26). A study (27) found that individuals’ perceptions of power and wealth influence the Cantril Ladder measure, suggesting that the measure may reflect socio-economic considerations. The Cantril Ladder effectively captures a global picture of subjective quality of life assessment. It differs in its meaning from other SWB instruments, such as the Satisfaction with Life Scale (SWLS). The SWLS measure uses a multi-item approach to assessing life satisfaction. Some researchers see this as potentially more reliable. The Positive and Negative Affect Scale (PANAS) measure, which captures emotional states rather than evaluative judgements, focuses on the affective components of perceived well-being. The Eurostat Statistics on Citizens’ Income and Living Conditions (EU-SILC) is an important indicator, especially in Europe. The survey includes questions on subjective life satisfaction supplemented with other indicators, including objective ones. EU-SILC measures provide a comprehensive picture of well-being in the European context (14, 28).

The measurement of subjective perception of well-being serves to identify sources of life satisfaction that are useful in formulating directions of socio-economic policy. The determinants of high subjective perception of well-being are of interest to socio-economic politicians and researchers who try to indicate the most effective actions to raise the subjective perception of well-being by ensuring universal health system and improving healthcare access (13).

The effectiveness of socio-economic policy depends on the precision of resource allocation (expenditures) to specific groups of recipients. The list of socio-economic policy tools includes increasing residents’ incomes, increasing investments in education and increasing the availability of social, health, educational, safety, housing, culture, sports and tourism services, caring for the quality of the natural environment, and child and senior care. Well-targeted interventions, comprising increasing incomes, ensuring affordable housing, expanding healthcare access, and enhancing education, contribute to both economic stability and well-being improvements. The subjective perception of individual well-being or the well-being of a household is shaped by the above-mentioned objective factors but corrected by multi-faceted comparisons with the situation of other people or households in the respondent’s immediate and distant environment. Incorporation of subjective well-being perception into socio-economic policy design can increase the efficiency of social and healthcare investments and maximise resource allocation successfully and equitably to improve overall quality of life.

A review of the literature on the efficiency of human development, with identification of 20 implementation gaps that offer potential for further research, is published in the article by E. Mariano et al. (29). Among these gaps, one of them highlights the use of DEA to construct indices or evaluate the efficiency of subjective well-being (SWB). Most existing studies concentrate solely on the economic aspect of human development. Although important, researchers still treat SWB as a relatively new and underexplored topic.

In recent years, the use of DEA in studies on subjective well-being (SWB) has become an increasingly popular method. Several studies use DEA to create composite indicators of quality of life. Among them are publications by (29–33). DEA is considered a tool for determining whether SWB is an economic production input or output (34). The results indicate that in most cases, SWB can be considered an input to production, but it is rarely an output in the sample of European countries. Closely related to well-being efficiency is the term “happiness efficiency.” It was created by M. Binder and T. Broekel (34) in their work on the ability of individuals to transform resources into SWB in Great Britain. Similarly, the work (35) contains an assessment of SWB efficiency in a group of 26 OECD member states. Based on the assumption that differences between individual countries result from social spending, unemployment rates, or institutional quality, the DEA method was used to examine the efficiency of Italian regions in transforming their inputs into SWB improvement (36).

In an extensive analysis of a set of 130 countries, the authors examined how four indicators of good governance, including technical quality of government, democratic quality of government, government consumption as a percentage of government consumption in total domestic consumption, government expenditure as a percentage of government expenditure in gross domestic product are translated into SWB growth (37). They used DEA as an analysis tool. In analogous work involving data from a group of 82 countries, authors examined efficiency while transforming resources such as income, education, and health into SWB (35). A new method, called stochastic semi-nonparametric data envelopment, was proposed in their analyses. The paper concludes that greater SWB efficiency is associated with higher social spending, civil liberties, quality of governance, and lower unemployment and inequality. Equally extensive research, with panel data from 91 countries, was used in a partial frontier approach (38). The authors found that higher SWB efficiency was associated with higher social support, freedom, rule of law, lower unemployment and forced part-time work.

In the work by F. Sarracino and K. O’Connor, the impact on SWB measured by the Cantril ladder was examined using the DEA method (17). The indicators described in the World Happiness Report, including real GDP per capita, healthy life expectancy, social support, freedom of choice, lack of corruption, and generosity, were analysed. Studies indicate that there is no correlation between SWB efficiency and economic efficiency. Moreover, countries with the highest SWB values, such as Nordic countries, did not have the highest SWB efficiency.

## 3 Materials and methods

**Figure 1:**
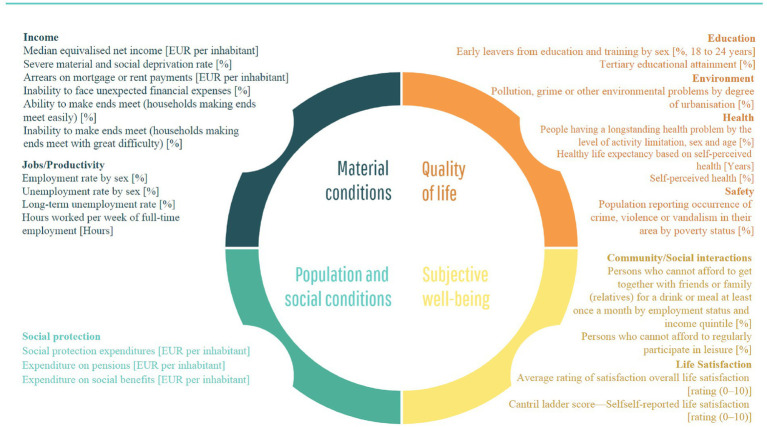
Domains and variables (indicators) used as proxies of well-being and quality of life.

Principal domains of well-being were the starting point of our considerations. Figure 1 juxtaposes well-being domains and their indicators. They may function as proxies for well-being. Selected variables are coherent with the literature of the subject, e.g., (5, 6, 16). There are four main domains of subjective well-being (54), i.e., material conditions, population and social conditions, quality of life, subjective well-being and overall experience of life. All listed indicators are variables from the Eurostat database.

### 3.1 Data

The analyses were based on the data from the EU-SILC and OurWorldInData surveys (66, 67). EU-SILC dataset consists of selected variables gathered for 2022 and chosen European countries, which were the subject of our analysis. Typical data imputation methods were applied to handle missing values. In case of a single missing value, using the mean of neighbouring observations was imputed. When missing boundary values, imputation was performed using regression-based estimates, ensuring the assumptions of ordinary least squares.

The dataset contains designated indicators of quality of living dimensions and well-being of dimensions (Tables 1, 2).

**Table 1:** Variables describing well-being and self-reported life satisfaction (2022) in European countries; *n* = 27.

Variables	Mean	Median	Min	Max	Q_1_	Q_3_	Std. dev.	Coef. var.
HHs making ends meet easily [%]	15.96	15.80	2.20	40.40	7.80	23.10	10.01	62.71
HHs making ends meet with difficulty [%]	6.93	6.00	1.40	36.80	2.90	8.40	6.80	98.04
Self-perceived health [%]	66.76	65.10	49.60	85.40	56.40	79.60	11.52	17.25
Healthy life expectancy [Year]	73.69	75.20	66.80	79.30	69.80	77.40	4.12	5.59
Overall life satisfaction [0–10]	7.13	7.10	5.50	7.80	6.90	7.50	0.48	6.67
Cantril ladder score [0–10]	6.63	6.59	5.47	7.80	6.21	6.91	0.56	8.38

**Table 2:** Variables describing material conditions, population and social conditions (2022) in European countries; *n* = 27.

Variables	Mean	Median	Min	Max	Q_1_	Q_3_	Std. dev.
Social protection expenditures total [EUR Mil.]	8,586.93	6,174.57	1,952.12	24,750.59	3,562.77	13,854.18	5,923.04
Expenditure on pensions [% of GDP]	10.64	10.00	4.50	16.40	8.10	13.40	3.18
Median income [EUR per inhabitant]	15,951.85^*^	18,472.00	9,671.00	33,214.00	12,277.00	20,941.00	5,892.54
Social protection benefits [EUR per inhabitant]	5,223.25^*^	6,046.13	1,889.44	24,358.92	3,486.87	13,315.25	5,706.32
Social protection Sickness/ Health care [Euro per inhab.]	1,542.56^*^	1,717.72	551.66	6,554.82	1,123.66	3,979.03	1,629.48
Long-term unemployment rate [%]	2.14	1.90	0.50	7.70	1.20	2.30	1.59
Employment rate [%]	76.38	77.50	64.80	82.90	74.00	80.20	4.96
Tertiary educational attainment [%]	44.82	44.40	24.70	62.30	37.10	51.40	9.89

*Harmonic mean.

Table 1 juxtaposes variables (indicators) used as well-being and quality of life proxies. Variables describe personal or household well-being and self-reported life satisfaction in 2022. The high value of the coefficient of variation (62.71%) of households that make ends meet easily and the even higher value for making ends meet with difficulty variable (98.04%) illustrates significant variability in EU countries. The mean overall life satisfaction in analysed countries was quite high (7.13, 0–10 scale). The minimum value was 5.5 (Bulgaria). A similar picture is with the *Cantril score*, where the minimal value was 5.47. The maximum (and minimum) value of the *Cantril score* and the maximum (and minimum) value of overall life satisfaction were almost identical. On the other hand, *Cantril score* was more concentrated around smaller values than overall life satisfaction (Q1 and Q3).

Table 2 contains basic descriptive statistics of variables describing material, population and social conditions. The range in the *Social protection expenditures total* variable is over 22,798, so there are significant differences between countries. Likewise between variable *Expenditures on pensions* [% of GDP] in the analysed countries there are substantial discrepancies. Moreover, the Median income [EUR per inhabitant] and the *Social protection benefits* [EUR per inhabitant] indicate considerable differences among countries. The variable *Social protection benefits* [EUR per inhabitant] have the highest value of the coefficient of variation (68.69%).

**Figure 2:**
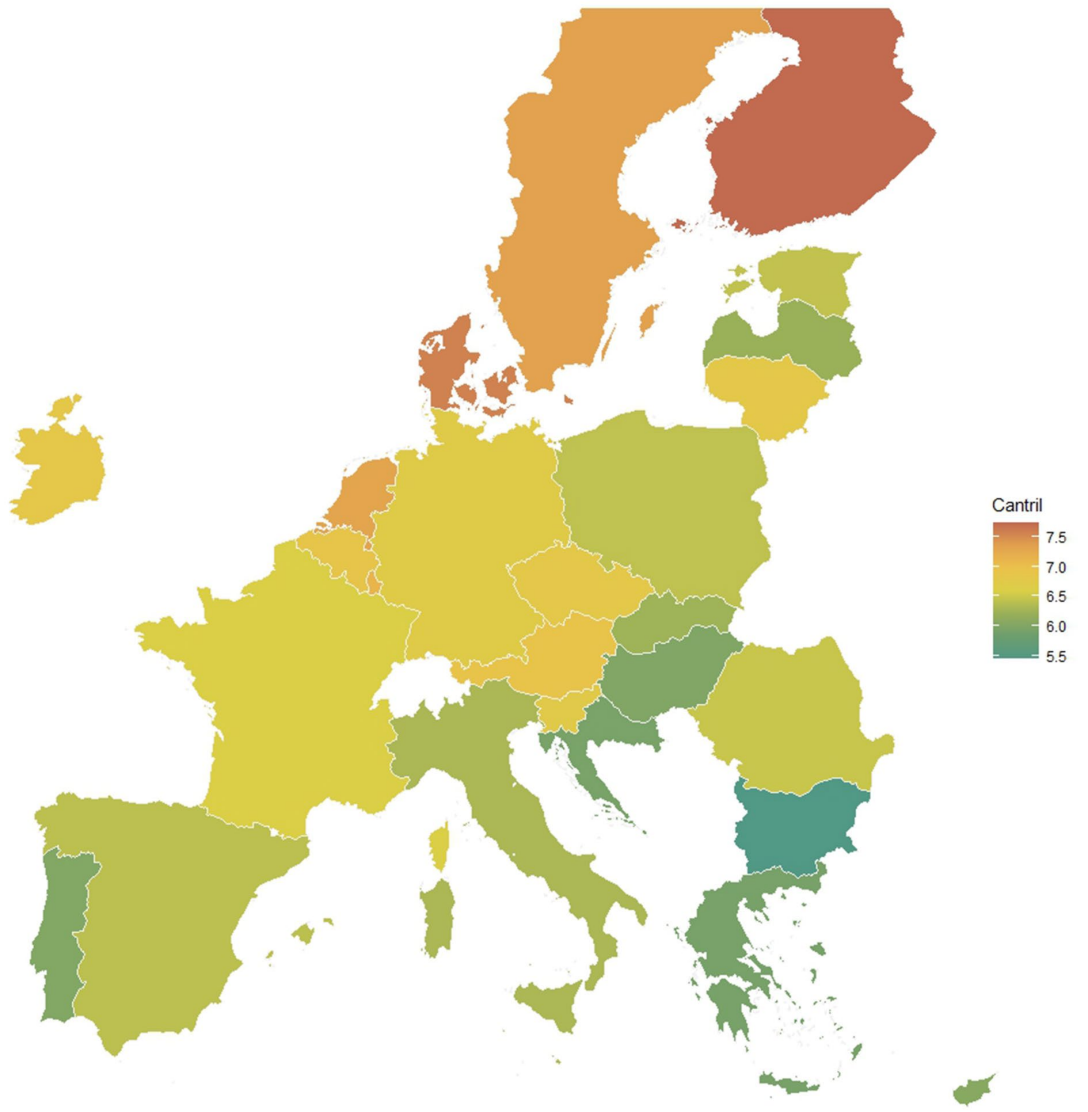
Map of well-being (27 European countries, Cantril ladder score 0–10, 2022).

The subjective well-being changes over time, but there is an increasing tendency overall (39). Figure 2 shows the mean values of the Cantril ladder score in 2022. Despite intensively pursuing the EU countries for the last few years, Bulgaria has the lowest level of well-being assessment. Most of the countries of the North have noticeable higher-than-average levels of the Cantril life ladder.

Our further considerations focus on the relation between variables: *Average rating of satisfaction and overall life satisfaction* [rating (0–10); symbol: E005], *Cantril ladder score* [rating (0–10); symbol: Cantril]; as well as variable: *households making ends meet easily* [%; symbol: E001] with other variables. The first set will be named “input variables,” and the rest of the variables “output variables.”

By and large, the correlations between pre-selected input and output variables were positive in our sample of European Countries. All variables that turn out negatively associated must not be included in the DEA model. The final preliminary set of variables for subsequent analysis encompasses variables: *Social protection benefits* [Euro per inhabitant; symbol: N0011], *Social protection: sickness/healthcare* [Euro per inhabitant; symbol: N0012], *Social protection expenditures sickness/healthcare and disability* [Euro per inhabitant; symbol: N0017], *Social protection expenditures Old age and survivors* [Euro per inhabitant; symbol: N0018], *Median equivalised net income* [Euro per inhabitant; symbol: N003], *Tertiary educational attainment* [percentage; symbol: X006] as potential input variables. Listed factors depict public outlays on healthcare, disability support, and elder care, which are essential to ensure healthy lives and promote well-being perception. To depict the role of economic stability and education in promoting long-term health and well-being, two additional measures were used: Median equivalised net income and Tertiary educational attainment.

**Figure 3:**
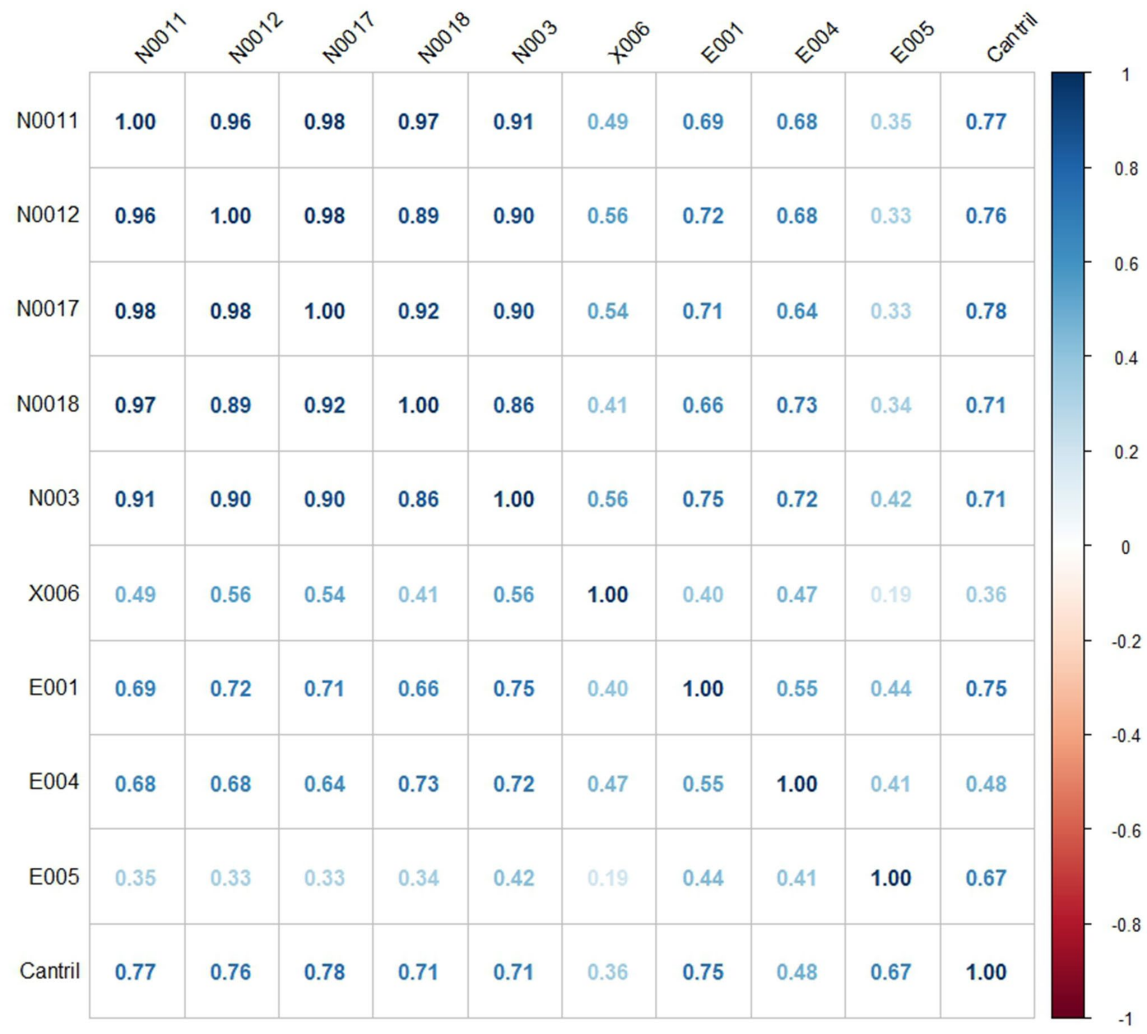
Correlation matrix (27 European countries, 2022).

The role of listed output factors is to capture the level of subjective well-being, financial security, and overall quality of life. And thus functioning as proxy for broader impact of effective healthcare and social protection policies. Variables: *Households making ends meet easily* [percentage; symbol: E001], *Overall life satisfaction* [rating (0–10); symbol: E005], and *Cantril ladder score* [rating (0–10); symbol: Cantril]; were possible output factors, under the condition that variables were not negatively correlated with some of the input variables in the sample of all 27 European Countries (Figure 3).

### 3.2 Classification of European countries

The input based classification of countries was applied to deepen insight into the problem of subjective well-being. The hierarchical clustering was chosen because it does not require a prior determination of the number of clusters. Therefore, in the classical Ward linkage procedure, objects’ similarity is determined based on increased squared error (40).

The procedure of hierarchical agglomeration is widely discussed in the literature (41–43). The data was processed in R by hclust (44).

As mentioned, we implemented two classification approaches. First, a multivariate clustering based on “input variables” (N003, X006, N0011, N0012, N0017 and N0018) resulted in homogenous groups of European countries in terms of expenditures on social policy. The task was to include factors that foster education awareness and accessibility to healthcare services; support enhancing economic stability and access to healthcare. A special emphasis was placed on factors ensuring health security and health coverage. This classification is based on the following “input variables”:

- *Median equivalised net income* [Euro per inhabitant; symbol: N003].
- *Tertiary educational attainment* [Percentage; symbol: X006].
- *Social protection: benefits* [Euro per inhabitant; symbol: N0011].
- *Social protection: sickness/healthcare* [Euro per inhabitant; symbol: N0012].
- *Social protection: sickness/healthcare and disability* [Euro per inhabitant; symbol: N0017].
- *Social protection expenditures: old age and survivors* [Euro per inhabitant; symbol: N0018].

The second classification was based on “output variables” (E001, E005 and Cantril) and resulted in similar groups of European countries concerning proxies of subjective well-being. Included factors represent subjective, s*elf-reported assessments based on individual perception of* financial stability elements which contribute to stress reduction and better mental health. This classification is based on the following “output variables”:

- *Ability to make ends meet (households making ends meet easily)* [Percentage; symbol: E001].
- *Cantril ladder score* [rating (0–10); symbol: Cantril].
- *Self-reported life satisfaction* [rating (0–10); symbol: E005].

The number of clusters in both classifications was determined by visually examining the obtained dendrograms, ensuring a data-driven approach to analysing the relationships between social spending, health, and well-being. Thus, division was when the distance between merged clusters increased significantly (2, 3). The second criterion of division into clusters was the statistical method based on the silhouette coefficient value (41).

### 3.3 Analysis of efficiency

Data Envelopment Analysis (DEA) is one of the most widespread methods for assessing the efficiency of decision-making units (DMUs). It is based on the best practice frontier concept. It assumes that all units in a given system can operate at a certain level of efficiency determined by efficient units (45).

Each DMU uses N different inputs: $x_{1},x_{2},\ldots ,x_{N}$ to obtain M different outputs (effects): $y_{1},y_{2},\ldots ,y_{M}$ . Let $x_{\mathrm{nj}}$ , n=1,2,…,N , j=1,2,…,J and $y_{\mathrm{mj}}$ , m=1,2,…,M , j=1,2,…,J be, respectively, the amount of n -th input and m -th output used (obtained) by ${\mathrm{DMU}}_{j}$ . It’s easy to perceive that for every $D_{s}\subset D$ and for every ${\mathrm{DMU}}_{i}\in D_{s}$ we have that if ${\mathrm{DMU}}_{i}$ is efficient in D (i.e., ${\mathrm{DMU}}_{i}$ ’s efficiency score obtained by DEA of D equals 1) then ${\mathrm{DMU}}_{i}$ is also efficient in $D_{s}$ and this is true for input- and output-oriented DEA. That means that it is justified to perform only DEA on the pairwise disconnected subsets $D_{s}\subset D,s=1,2,\ldots ,S$ instead of a whole set D , which allows us to perform analysis only on units that are, for example, similar to each other, which may be better than such analysis performed on the whole set D . In such cases, benchmarks for non-efficient units should be more achievable.

One of the most significant disadvantages of the DEA method is redundancy: the number of efficient units in the analysed set is often much larger than the total number of units (46, 47). In the literature, there are several modifications of DEA that can prevent redundancy (48–50). B. Guzik introduced the non-profit efficiency approach, known as the NPE method (47). The author proposed replacing the actual values of inputs and outputs with valuations of their units. Valuations are carried on the entire system of DMUs, and not on individual values. The NPE method was originally intended to assess the efficiency of non-profit entities. Nevertheless, it can be used for a broader range of problems (the author proposed a modification of NPE that can be used for profit-oriented entities).

Let $a_{n}$ , n=1,2,…,N and $b_{m}$ , m=1,2,…,M be the unit valuation of n -th input and m -th output, respectively. These valuations are unknown; one must find them to calculate each unit’s efficiency score. Let also $A_{j}=\sum_{n=1}^{N}a_{n}x_{\mathrm{nj}}$ and $B_{j}=\sum_{m=1}^{M}b_{m}y_{\mathrm{mj}}$ be equal to the total values of inputs and outputs of ${\mathrm{DMU}}_{j}$ , respectively. Then $A=\sum_{j=1}^{J}A_{j}$ and $B=\sum_{j=1}^{J}B_{j}$ are total values of inputs and outputs in the whole system. The efficiency score equals B/A , where both are obtained by solving the linear programming task below. Maximise B−A

under constraints:


(1) $$B_{j}-A_{j}\leq 0\;\text{for each}\;j=1,2,\ldots ,J$$

(2) $$\sum_{m=1}^{M}b_{m}=1$$

(3) $$b_{m}\geq 0\;\text{for each}\;m=1,2,\ldots ,M$$

The most significant advantage of the NPE method is a smaller number of efficient DMUs, compared to DEA, and the requirement to solve only one linear programming task at a time (in DEA, we need to solve tasks for each DMU), which makes it simpler and faster than DEA. Moreover, this method can be applied to the efficiency analysis of small sets of DMUs (few in the number of elements). Since the NPE method was developed only for input-oriented efficiency analysis, we propose its modification, which may be called output-oriented NPE. Here, A and B are obtained by solving the task below. Maximise B−A under constraints:


(4) $$B_{j}-A_{j}\leq 0\;\;\text{for each}\;j=1,2,\ldots ,J$$

(5) $$\sum_{n=1}^{N}a_{n}=1$$

(6) $$a_{n}\geq 0\;\;\text{for each}\;n=1,2,\ldots ,N$$

The NPE uses an approach where the researcher takes a new unit, “artificial” in some sense, for which the level of each input equals the sum of the levels of this input for every unit in the analysed system. Solving the problem is similar to input-oriented DEA, but instead of maximising the ratio of used inputs and outputs, maximise their difference. The efficiency score of each unit is then calculated as the ratio of the valued output of this unit and its valued input. Similarly, we designed a modified NPE method, which is based on output-oriented DEA in the same way as NPE is based on input-oriented DEA.

## 4 Results

The first part of our two-pronged study of factors determining well-being was to identify homogenous groups of European Union member countries, similar in terms of social policy expenditures (“input variables” approach) and subjective well-being (“output variables” approach). The studied set of countries is heterogenic, and the sample countries were grouped to discover whether there is a change in the magnitude of the associations into homogeneous groups.

As a result of the first classification, we got three homogenous groups of European countries similar in terms of expenditures on social policy. The socio-economic discrepancies between countries are slighter inside the determined clusters than in the entire EU. The first group of countries consists of 10 northern and central European countries. The second group was the most numerous; the third group was the smallest—five countries. Additionally, notably that this classification gave groups of countries that were closely geographically located as well, with few exceptions, e.g., Portugal, Bulgaria, and Cyprus.

**Figure 4:**
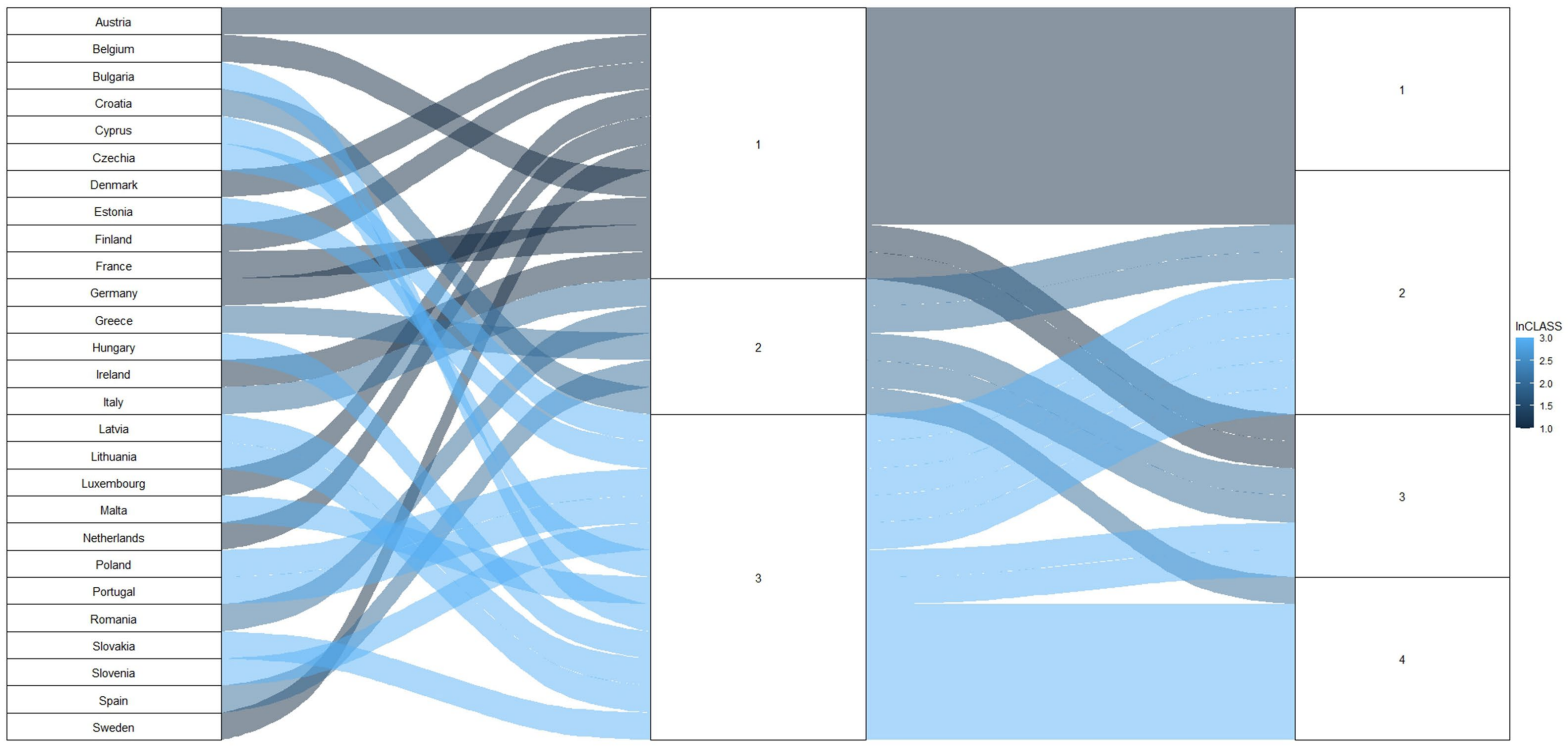
Classifications outcomes: based on “input variables” (three clusters) and based on “output variables” (four clusters).

Classification based on “output variables” resulted in four homogenous groups of European countries concerning subjective well-being variables. Consequently, countries in respective clusters have comparable levels of subjective well-being. The three clusters are approximately the same size, with one slightly larger (Figure 4). Moreover, the first cluster includes all countries that belong to the first cluster based on the “input variables” classification. Therefore the first classes (named “Mint”) in both classifications consist of Denmark, Luxembourg, the Netherlands, Austria, Finland, and Sweden, countries that are similar in terms of input and output variables (Figure 4).

### 4.1 Classification based on “input variables”

**Figure 5:**
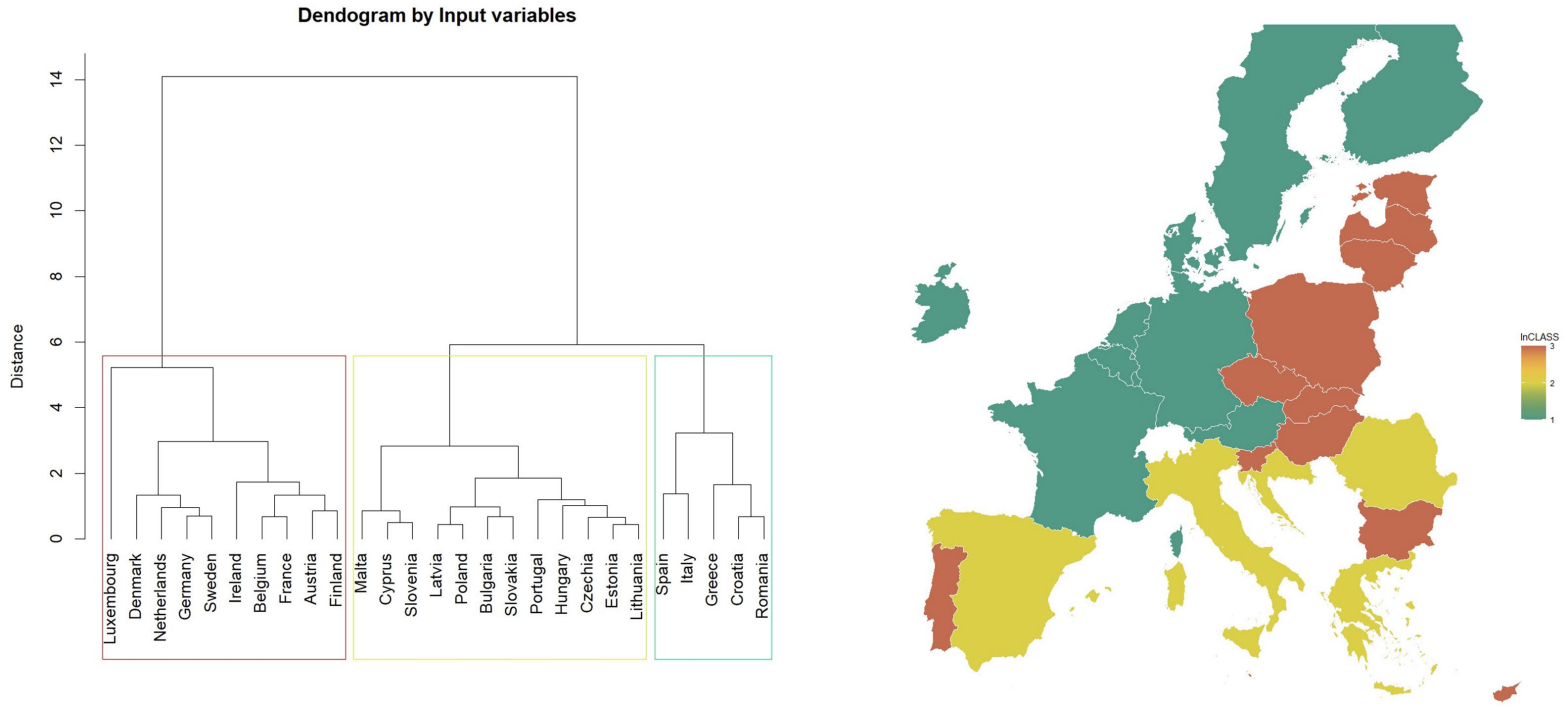
Map of classification based on “input variables” and dendrogram (27 European countries, three clusters) in 2022.

The dendrogram (Figure 5) suggests that the European countries can be divided into three clusters in the first classification. The division is also confirmed by the value of the silhouette index (68). The class named “Mint” includes a large portion of Western and Northern Europe, including countries like Luxemburg, Denmark, the Netherlands, Germany, Sweden, Ireland, Belgium, France, Austria, and Finland. These countries tend to have high values for most variables (Figure 5). Luxemburg presents the highest values for most variables.

**Figure 6:**
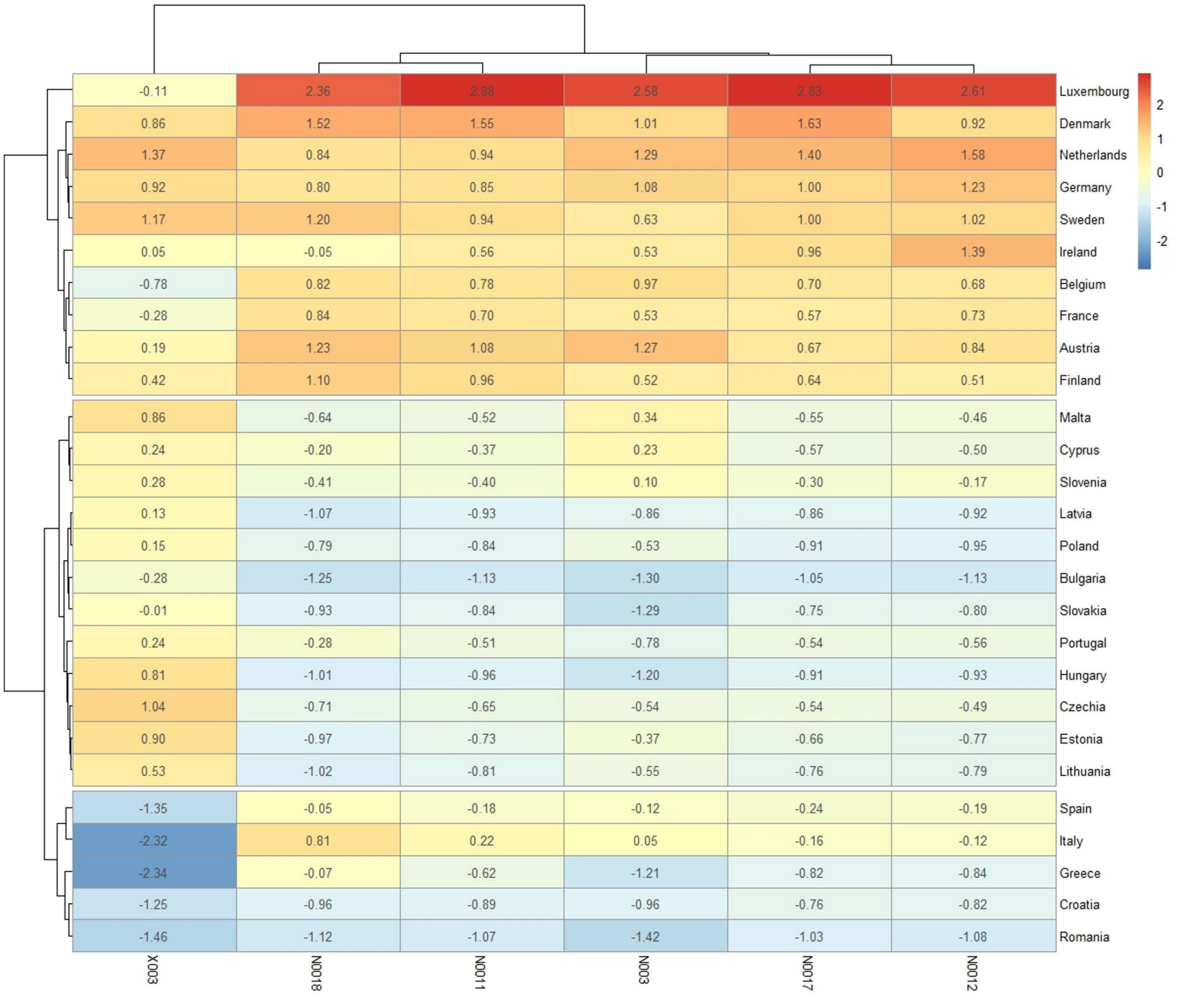
Classifications based on “input variables” (heatmap for three clusters) in 2022, standardised variables.

Figure 6 shows groups of both: rows (countries) and columns (variables) of the matrix of standardised values, revealing patterns in three distinguished clusters. The “Red” class primarily includes Eastern European countries, plus Portugal, Malta and Cyprus. This is the most numerous group, with the countries that tend to have lower values for most of the variables used for classification (e.g., N018, N011, N017 and N012). The “Yellow” class contains Spain, Italy, Greece, Croatia, and Romania. It is a group with the most diverse ranges of values. Some variables have very low values (e.g., X003).

### 4.2 Classification based on “output variables”

**Figure 7:**
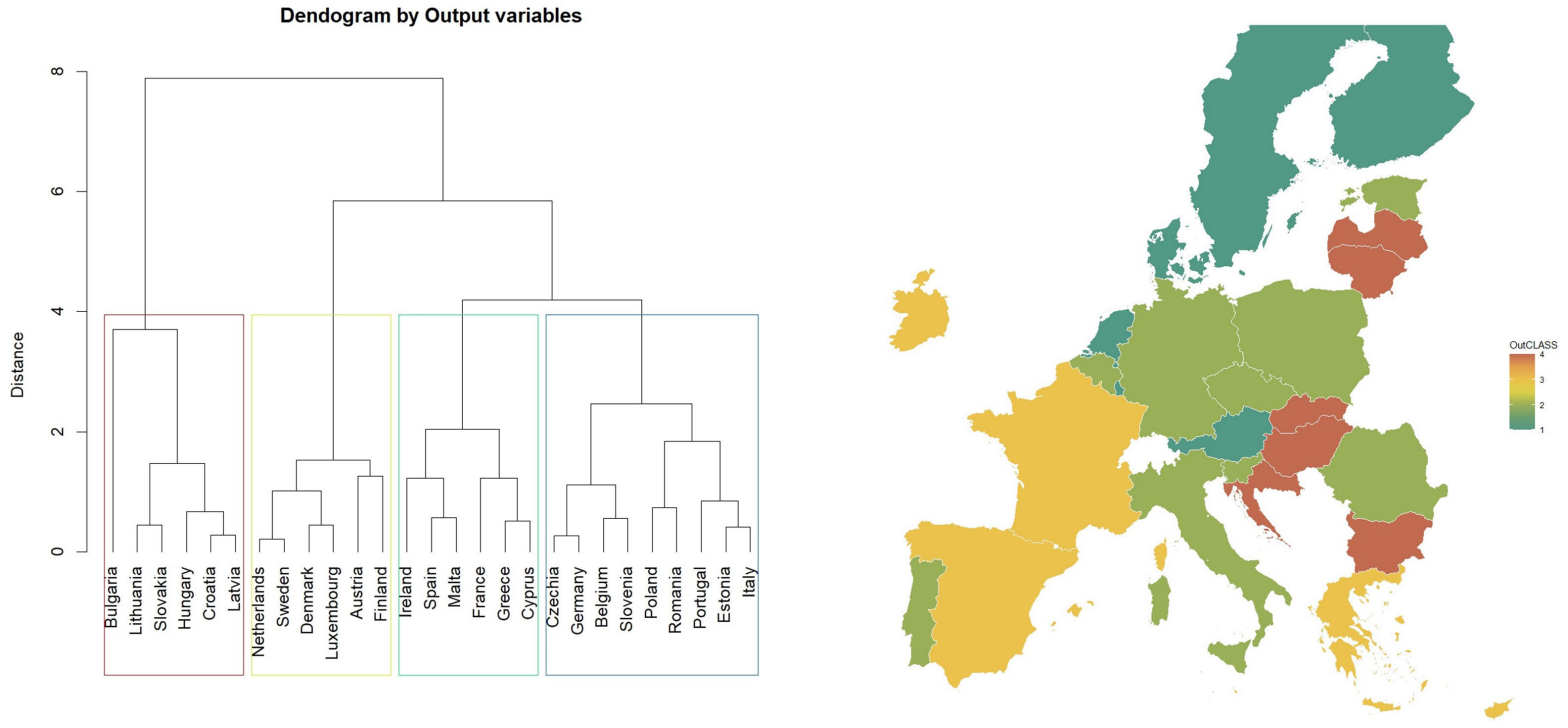
Map of classification based on “output variables” and dendrogram (27 European countries, four clusters) in 2022.

As a result of the second classification, European Union countries were divided into four clusters. This indication is based on visually examining the obtained dendrogram (Figure 7). The value of the silhouette index also supported this decision.

The class labelled “Mint” mainly contains Northern European countries, including Sweden, Denmark, Finland, and additionally the Netherlands, Austria, and Luxemburg. Countries in the “Mint” class manifest high values for most variables (Figure 7), especially Finland and Austria. In Figure 7, groups for rows (countries) and columns (variables) are shown based on the matrix of standardised values in four clusters.

**Figure 8:**
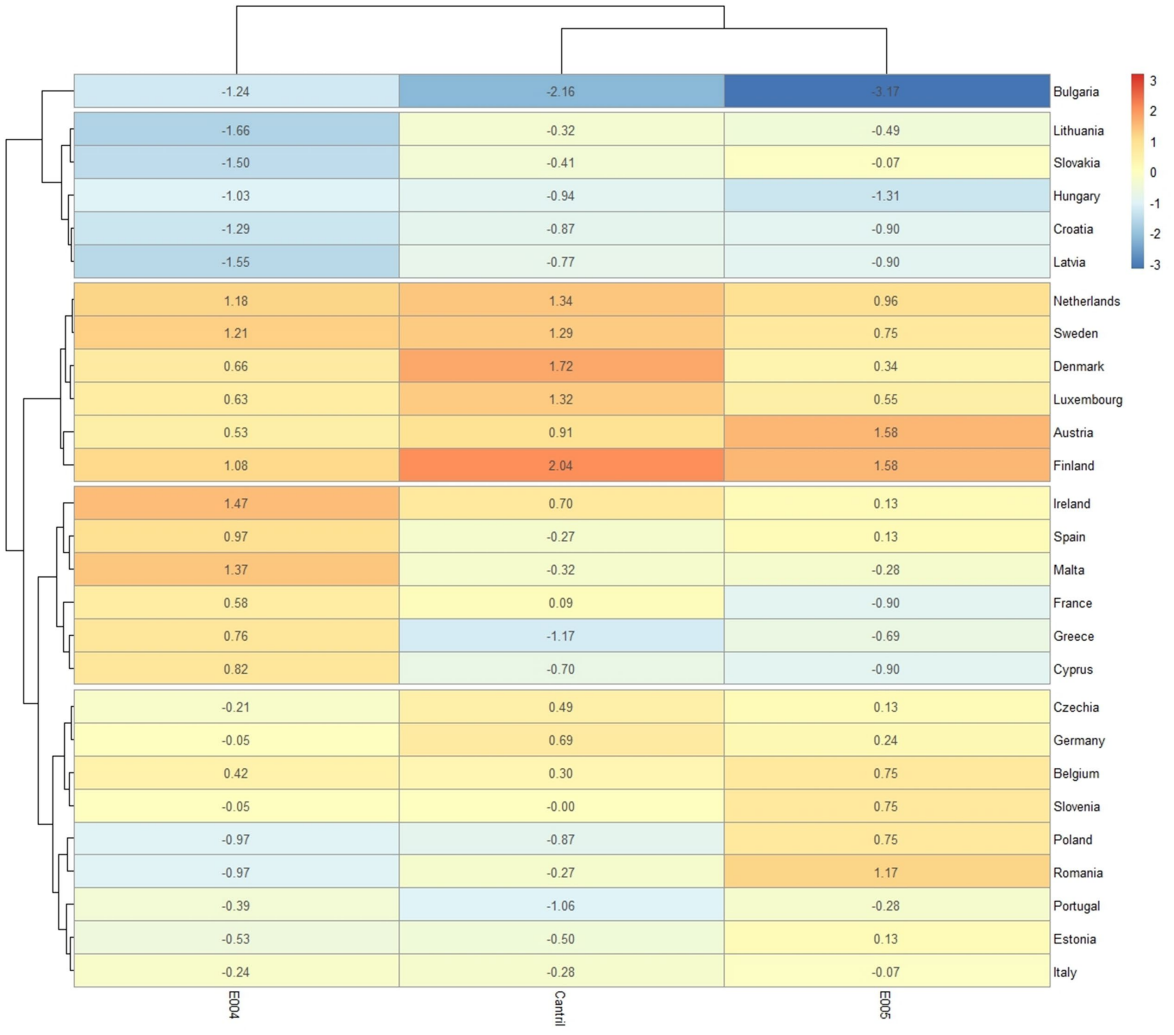
Classifications based on “output variables” (heatmap for four clusters), standardised variables.

The “Red” class includes the countries of Eastern Europe. This group contains countries with lower values for most of the variables used for clustering: E004, E005, and Cantril. The “Yellow” class contains Ireland, Spain, Malta, France, Greece, and Cyprus. It is a group with the most diversified values—some variables have significantly higher values (e.g., Cantril, E005). The “Green” class: Czechia, Germany, Belgium, Slovenia, Poland, Romania, Portugal, Estonia, and Italy, is a group with a low range of values, especially variables E004 and Cantril (Figure 8).

## 5 The data envelopment analysis and non-profit efficiency (2022)

Both DEA and NPE analysis require each input variable that will be used for analysis to be positively correlated with each output variable. As a result of the examination of correlations, five input and two output variables were chosen. The inputs taken to the model were N003 (*Median equivalised net income*), X006 (*Social protection*: benefits), N0012 (*Social protection: sickness/healthcare*), N0017 (*Social protection: sickness/healthcare and disability*) and N0018 (*Social protection: old age and survivors*). The output variables taken to the model were E001 (*Ability to make ends meet*) and Cantril (*Cantril ladder score*). The excluded input variables do not depict social policy, nor are the variables taken into account in the model. Both employment rate and tertiary education attainment depend on expenditures on social policy, but the government cannot control them to the same extent as expenditures.

We expected recommendations for the non-efficient units in both the DEA and NPE models. In input-oriented models, these recommendations inform at which levels of input every unit should work to guarantee the same results and be efficient. Because of that, this orientation was used for DEA and NPE models in classification based on “output” variables, since these are very similar in all countries from the same group. An analogous approach was used for classification based on “input” variables, where levels of inputs were analogous within each group. In this case, we used output-oriented models since we obtained information on which each non-efficient unit should get an output level to be efficient at the actual input level.

### 5.1 DEA and NPE output-oriented for the first classification based on “input variables”

For the classification of units based on “input variables,” the output-oriented group DEA and output-oriented group NPE methods were used. The analysis results indicate the level of output at which units are considered efficient under *ceteris paribus* for input variables. The results of DEA and NPE analyses are presented in Table 3.

**Table 3:** Results of group DEA and NPE analysis for classification based on “input variables.”

Country	Group	DEA score	DEA rank	NPE score	NPE rank
Ireland	Mint	1.0000	1	1.0000	1
Netherlands		1.0000	1	0.8644	4
Finland		1.0000	1	1.0000	1
Belgium		0.9882	2	0.8630	5
Sweden		0.9645	3	0.9434	2
Germany		0.9589	4	0.8593	6
France		0.9547	5	0.9165	3
Denmark		0.8918	6	0.8237	7
Austria		0.8610	7	0.8115	8
Luxembourg		0.5878	8	0.5521	9
Spain	Yellow	1.0000	1	0.4545	4
Romania		1.0000	1	0.6271	2
Greece		0.8330	2	1.0000	1
Croatia		0.7597	3	0.5167	3
Italy		0.5388	4	1.0000	1
Poland	Red	1.0000	1	0.7580	7
Estonia		1.0000	1	1.0000	1
Hungary		1.0000	1	0.9542	2
Bulgaria		1.0000	1	0.5777	11
Czechia		1.0000	1	1.0000	1
Slovakia		1.0000	1	0.7558	8
Slovenia		0.9938	2	0.9512	3
Latvia		0.9549	3	0.7684	6
Lithuania		0.9537	4	0.7138	9
Portugal		0.9279	5	0.8777	4
Cyprus		0.8461	6	0.8395	5
Malta		0.6593	7	0.6119	10

The NPE analysis significantly reduced redundancy in the “Red” group. In DEA, 6 of 12 countries obtained maximal efficiency score: Poland, Estonia, Hungary, Bulgaria, Czechia, Slovakia and Slovenia. That means that, in DEA, in all these countries expenditures for social policy were used efficiently. According to NPE analysis, social policies were implemented efficiently only in two countries, which were also efficient in DEA: Estonia and Czechia. Also, Hungary, one of among efficient countries in DEA, obtained a very high efficiency score in NPE analysis—its social policy is implemented in the best way from all NPE-non-efficient units. On the other hand, one of the DEA-efficient countries in this group, Bulgaria, was the least NPE-efficient. A similar situation occurred with two other DEA-efficient countries: Slovakia, which was the fourth least NPE-efficient and Poland, which was the fifth least NPE-efficient country. In both models, one of the least efficient countries in the “Red” group was Malta—the least DEA-efficient and second least NPE-efficient.

The most significant differences between the results of DEA and NPE analyses occurred in the “Yellow” group. None of the DEA-efficient countries (Spain, Romania) was NPE-efficient (Greece, Italy) and vice versa. Moreover, Spain was the least NPE-efficient, and Italy was the least DEA-efficient. One can also see that all countries whose social policies were not NPE-efficient obtained quite poor NPE-efficiency scores (although Romania, which was DEA-efficient, has the highest score of all non-NPE-efficient countries).

In the “Mint” group, the differences between NPE and DEA were least significant. In both models the social policies in Ireland and Finland were efficient (and the Netherlands in DEA). The least efficient social policy was that implemented by Luxembourg, which obtained very poor both DEA- and NPE-efficiency scores.

Using the DEA method, one can also obtain benchmarks for non-efficient units. These benchmarks inform which countries can be role models for non-efficient ones when they decide to change their social policies. The NPE method is that such benchmarks cannot be obtained directly from the NPE task; approximate benchmarks can be obtained using approximation methods. For our analysis, the OLS method was applied. The results of benchmark analyses are introduced in Table 4. The larger the value of “lambda” is, the more efficient unit should be as a role model for the non-efficient one.

**Table 4:** Group benchmarks in output-oriented NPE and DEA (“input variables”).

Country	Group	DEA benchmarks and lambdas	NPE benchmarks and lambdas
Ireland	Mint	Ireland (1.0000)	Ireland (1.0000)
Netherlands		Netherlands (1.0000)	Ireland (1.0253), Finland (0.2094)
Finland		Finland (1.0000)	Finland (1.0000)
Belgium		Ireland (0.0490), Netherlands (0.3603), Finland (0.5042)	Ireland (0.0874), Finland (1.0068)
Sweden		Finland (0.9824)	Finland (1.0035)
Germany		Ireland (0.0441), Netherlands (0.5746), Finland (0.3368)	Finland (1.1533)
France		Ireland (0.2007), Netherlands (0.1482), Finland (0.5758)	Ireland (0.2382), Finland (0.7386)
Denmark		Netherlands (0.0751), Finland (1.0188)	Finland (1.1607)
Austria		Ireland (0.0424), Finland (1.0187)	Ireland (0.4354), Finland (0.7511)
Luxembourg		Ireland (0.1316), Finland (1.4591)	Ireland (0.8492), Finland (0.5294)
Spain	Yellow	Spain (1.0000)	Italy (0.2664), Greece (1.0901)
Romania		Romania (1.0000)	Greece (0.8183)
Greece		Romania (1.0805)	Greece (1.000)
Croatia		Romania (1.2237)	Greece (1.0305)
Italy		Spain (0.0501), Romania (1.7549)	Italy(1.0000)
Poland	Red	Poland (1.0000)	Czechia (0.5319), Estonia (0.4057)
Estonia		Estonia (1.0000)	Estonia (1.0000)
Hungary		Hungary (1.0000)	Czechia (0.8825)
Bulgaria		Bulgaria (1.0000)	Czechia (0.7985)
Czechia		Czechia (1.0000)	Czechia (1.0000)
Slovakia		Slovakia (1.0000)	Czechia (0.9450)
Slovenia		Estonia (1.1013)	Czechia (1.2468)
Latvia		Bulgaria (0.5397), Estonia (0.2622), Hungary (0.3085)	Czechia (0.9076)
Lithuania		Bulgaria (0.8389), Estonia (0.3012), Hungary (0.0929)	Czechia (0.9880)
Portugal		Czechia (0.3675), Hungary (0.6482)	Czechia (0.8718)
Cyprus		Estonia (1.1556)	Czechia (1.2014), Estonia (0.0730)
Malta		Bulgaria (0.0468), Estonia (0.5404), Hungary (0.6750), Poland (0.2770)	Czechia (0.3447), Estonia (0.9021)

The analysis indicates that for most countries, benchmarks consisted of efficient countries that are closest to them geographically or culturally. For example, in the “Yellow” group, there were two countries (Spain and Romania) where social policy was implemented DEA-efficiently. The analysis demonstrates that Spain serves as a benchmark exclusively for Italy. For Croatia and Greece, only Romania was the benchmark. The same goes for the NPE analysis for the “Yellow” group: Greece was the only benchmark for Romania and Croatia. A similar situation occurred in the “Mint” group: in NPE analysis, Finland was the only benchmark for each Scandinavian country.

In cases of countries for which the property described above does not hold, usually countries that are neighbours have similar (or even the same) benchmarks. Finland turned out to be the benchmark for Germany, France, Austria and Luxembourg. For Latvia and Lithuania in DEA, Bulgaria is the main benchmark.

In the “Mint” group, Finland should be considered as a role model for every country where social policy is not implemented efficiently, regardless of whether the DEA or NPE method for efficiency analysis is used. Finland is the only country which has such a feature. A few countries have very similar properties, just for one analysis method. For the NPE method, such countries are Greece (the benchmark for all non-efficient units in the “Yellow” group) and Czechia (the benchmark for all non-efficient units in the “Red” group). For the DEA method, this feature holds for Romania, which can be considered a role model for all non-efficient units in the “Yellow” group. Estonia is also the benchmark for all but one (that is Portugal) units in the “Red” group.

An important feature may be called “benchmark preservation.” This term is defined as follows, under the assumption that unit A is the benchmark for non-efficient unit B in the DEA model. Let A be efficient and B be non-efficient in the NPE model. Then A is the benchmark for B in the NPE model. Thus, being a benchmark in the DEA model causes the unit to be a benchmark in the NPE model. This does not hold in the other way, being a benchmark in the NPE model does not cause being a benchmark in the DEA model.

Knowing benchmarks and lambdas for each non-efficient unit, one may obtain target values of outputs for each of them, i.e., values of outputs that each non-efficient unit should receive, with inputs not changed, to be efficient. Table 5 contains actual and target values of all output variables for each country with non-efficient social policy, both in DEA and NPE models. Since the NPE model targets and benchmarks were only approximated, achieving targets does not mean that a non-efficient country will become efficient—it will become only as close to efficient as approximation allows.

**Table 5:** Targets in group DEA and NPE analysis for classification based on “input variables.”

Country	Group	E001 actual	Cantril actual	E001 DEA target	Cantril DEA target	E001 NPE target	Cantril NPE target
Ireland	Mint	11.60	6.91	11.60	6.91	11.60	6.91
Netherlands		40.40	7.40	40.40	7.40	17.63	8.72
Finland		27.40	7.80	27.40	7.80	27.40	7.80
Belgium		28.60	6.86	28.94	6.94	28.60	8.46
Sweden		25.90	7.40	26.92	7.67	27.50	7.83
Germany		31.60	6.89	32.95	7.19	31.60	9.00
France		23.00	6.66	24.09	6.98	23.00	7.41
Denmark		27.60	7.59	30.95	8.51	31.80	9.06
Austria		23.10	7.08	28.40	8.24	25.63	8.87
Luxembourg		17.10	7.23	41.50	12.30	24.36	10.00
Spain	Yellow	20.50	6.44	20.50	6.44	10.46	10.00
Romania		6.50	6.59	6.50	6.59	6.69	6.76
Greece		2.20	5.93	7.02	7.12	2.20	5.93
Croatia		5.20	6.13	7.95	8.06	5.50	6.43
Italy		6.70	6.40	12.43	11.89	6.70	6.40
Poland	Red	9.60	6.26	9.60	6.26	15.71	6.26
Estonia		18.00	6.46	18.00	6.46	18.00	6.46
Hungary		8.00	6.04	8.00	6.04	13.94	6.04
Bulgaria		2.40	5.47	2.40	5.47	12.62	5.47
Czechia		15.80	6.85	15.80	6.85	15.80	6.85
Slovakia		4.60	6.47	4.60	6.47	14.93	6.47
Slovenia		19.70	6.65	19.82	7.11	19.07	8.53
Latvia		8.10	6.21	8.48	6.51	14.34	6.21
Lithuania		7.80	6.76	8.18	7.09	15.61	6.76
Portugal		10.20	5.97	10.99	6.43	13.78	5.97
Cyprus		17.60	6.13	20.80	7.46	20.30	8.69
Malta		11.80	6.30	17.90	9.56	21.68	8.18

For most countries, both NPE and DEA methods gave targets for both outputs greater than actual. A noteworthy result was obtained for Luxembourg, in which the mean Cantril ladder score should exceed value 10 (in DEA) for Luxembourg to be efficient. That means that it is not possible for Luxembourg to efficiently implement its social policy without lowering the expenditures. An analogous conclusion can be formulated for Italy using the DEA model. Importantly that in DEA, both Luxembourg and Italy achieved the lowest efficiency scores in their groups. In the NPE model, Luxembourg should achieve a mean score value of the Cantril ladder equal to 10. This target value exceeds empirical feasibility as it requires that for all respondents this score should be equal to 10. The same impossible target was obtained using the NPE method for Spain. Also here, both Luxembourg and Spain were the least efficient in their groups.

For countries whose policies can be improved, the most significant change in the “Red” group is needed in the case of Malta. The percentage of households that make ends meet (E001 variable) should improve from 11.8 to almost 17.9, and the Cantril score should improve from 6.3 to 9.56. In the “Yellow” group, Greece’s and Croatia’s social policies need much improvement. In Greece, the variable E001 value needs to rise from 2.2 to 7.02 and in Croatia, 5.2 to 7.95. The Cantril score in Greece should increase from 5.93 to 7.12 and in Croatia from 6.125 to 8.06. Minor adjustments are needed in the “Green” group, except Luxembourg, where improvements are impossible. Austria needs to change the most: from 23.1 to 28.4 in the E001 and from 7.08 to 8.24 in the Cantril score.

According to the results of the NPE method, in the “Red” group, the most remarkable improvements are required in Bulgaria, which should increase the value of the E001 from 2.4 up to 12.62, which also looks impossible to reach in the foreseeable future. On the other hand, its Cantril score does not need any changes. A notable situation occurred in the “Yellow” group, where Spain not only should achieve a 10 in Cantril score, but also its value of the E001 variable is too high, given the expenditures. As for Romania and Croatia, the necessary adjustments are not so significant. In the “Mint” group, minor adjustments are needed. The high value of the E001 variable and low Cantril score for the Netherlands is an example of an unusual situation.

### 5.2 DEA and NPE input-oriented for the second classification based on “output variables”

Similar analyses from the previous section were performed for classification based on “output variables.” Each group of countries is similar concerning “output variables” this time, so the authors chose to perform input-oriented DEA and NPE analysis. In this case, we compare social politics, which provide similar, in some sense, results. So, the efficiency of such politics base on the right distribution of funds. The results of DEA and NPE efficiency analysis are presented in Table 6. Table 7 contains benchmarks for all analysed countries.

**Table 6:** Results of group DEA and NPE analysis for classification based on “output variables.”

Country	Group	DEA score	DEA rank	NPE score	NPE rank
Luxembourg	Mint	1.0000	1	1.0000	1
Netherlands		1.0000	1	1.0000	1
Denmark		0.9769	2	0.9769	2
Finland		0.8707	3	0.8707	3
Sweden		0.8632	4	0.8632	4
Austria		0.8069	5	0.8069	5
Czechia	Green	1.0000	1	1.0000	1
Estonia		1.0000	1	0.9804	2
Germany		1.0000	1	1.0000	1
Romania		1.0000	1	1.0000	1
Slovenia		0.9431	2	0.9322	3
Portugal		0.9209	3	0.9035	5
Belgium		0.9192	4	0.9095	4
Poland		0.8634	5	0.7798	6
Italy		0.5385	6	0.5108	7
Cyprus	Yellow	1.0000	1	0.9208	3
Greece		1.0000	1	1.0000	1
Malta		1.0000	1	1.0000	1
Spain		1.0000	1	1.0000	1
France		0.9409	2	0.7600	4
Ireland		0.9225	3	0.9225	2
Bulgaria	Red	1.0000	1	1.0000	1
Hungary		1.0000	1	1.0000	1
Latvia		1.0000	1	0.9972	2
Lithuania		1.0000	1	1.0000	1
Slovakia		1.0000	1	0.8666	3
Croatia		0.8366	2	0.8067	4

**Table 7:** Group benchmarks in output-oriented NPE and DEA (“output variables”).

Country	Group	DEA benchmarks and lambdas	NPE benchmarks and lambdas
Luxembourg	Mint	Luxembourg (1.0000)	Luxembourg (1.0000)
Netherlands		Netherlands (1.0000)	Netherlands (1.0000)
Denmark		Luxembourg (0.6176), Netherlands (0.4217)	Luxembourg (0.5243), Netherlands (0.5129)
Finland		Luxembourg (0.6798), Netherlands (0.3905)	Luxembourg (0.5391), Netherlands (0.5279)
Sweden		Luxembourg (0.6470), Netherlands (0.3672)	Luxembourg (0.5114), Netherlands (0.4996)
Austria		Luxembourg (0.6996), Netherlands (0.2758)	Luxembourg (0.4913), Netherlands (0.4790)
Czechia	Green	Czechia (1.0000)	Czechia (1.0000)
Estonia		Estonia (1.0000)	Czechia (0.5346), Germany (0.2740), Romania (0.1377)
Germany		Germany (1.0000)	Germany (1.0000)
Romania		Romania (1.0000)	Romania (1.0000)
Slovenia		Czechia (0.2262), Germany (0.1535), Estonia (0.6264)	Czechia (0.3005), Germany (0.4201), Romania (0.2576)
Portugal		Germany (0.1739), Romania (0.7239)	Czechia (0.2776), Germany (0.0726), Romania (0.5414)
Belgium		Germany (0.7282), Estonia (0.3104)	Czechia (0.1829), Germany (0.8136)
Poland		Estonia (0.2944), Romania (0.6616)	Czechia (0.2940), Germany (0.0309), Romania (0.6123)
Italy		Germany (0.0154), Romania (0.95587)	Czechia (0.0422), Romania (0.9281)
Cyprus	Yellow	Cyprus (1.0000)	Spain (0.7245), Malta (0.2329)
Greece		Greece (1.0000)	Greece (1.0000)
Malta		Malta (1.0000)	Malta (1.0000)
Spain		Spain (1.0000)	Spain (1.0000)
France		Spain (1.1220)	Spain (1.1141)
Ireland		Greece (0.3674), Spain (0.2284), Malta (0.5177)	Greece (0.4438), Spain (0.3090), Malta (0.3635)
Bulgaria	Red	Bulgaria (1.0000)	Bulgaria (1.0000)
Hungary		Hungary (1.0000)	Hungary (1.0000)
Latvia		Latvia (1.0000)	Lithuania (0.1101), Hungary (0.9052)
Lithuania		Lithuania (1.0000)	Lithuania (1.0000)
Slovakia		Slovakia (1.0000)	Bulgaria (0.7749), Lithuania (0.1873), Hungary (0.1599)
Croatia		Bulgaria (0.4085), Hungary (0.3920), Slovakia (0.2357)	Bulgaria (0.5474), Lithuania (0.2272), Hungary (0.2642)

The analysis reveals that geographically proximate countries demonstrate similar benchmarks. Moreover, similarly to classification based on “output variables,” the property of “benchmark preservation” also occurs. In the “Red” group, the redundancy of efficiency significantly decreased when the NPE method was used instead of DEA. In the DEA, only one country’s (out of six) social policy was non-efficient; this country was Croatia. In the NPE analysis, two more countries, Latvia and Slovakia, also obtained efficiency scores that were smaller than one. However, in both methods, Croatia’s social policy was implemented in the most non-efficient way.

For classification based on “output” variables, efficiency rankings in almost all groups were very similar. The only significant difference is France, which was the best of the non-efficient countries in the “Yellow” group, according to DEA, with quite a high efficiency score of 0.9409 and worst of non-efficient countries, according to NPE, with a much lower efficiency score 0.7900. Italy stands out the most from the rest of the countries in its group in terms of both methods. In the DEA method, Italy obtained a poor efficiency score of 0.5385; the second lowest efficiency score was Poland’s 0.8634. In the NPE method, Italy obtained an even poorer efficiency score of 0.5108, but the second lowest efficiency score was closer to it (although still quite far)—again it was Poland with a score of 0.7798. Recommendations can only cover changes in the values of input variables because both DEA and NPE models are input-oriented.

### 5.3 Results and interpretation

The Northern European group (Mint), both in input- and output-based classifications, includes the most efficient countries. The output-oriented DEA and NPE models for the input-based classification confirm the high efficiency of Austria, Denmark, Finland, Luxembourg, the Netherlands and Sweden. The country that consistently emerges as the most robust benchmark across both models is Finland, the only country that serves as a benchmark for all non-efficient DMU within the Mint cluster (in DEA and NPE). Austria manifests a high level of efficiency as well. According to the DEA model, Austria should increase its Cantril score from 7.08 to 8.24 and the share of households that make ends meet easily from 23.1 to 28.4%. These countries demonstrate how comprehensive and well-targeted social protection systems can effectively convert public expenditures into subjective well-being.

Estonia stands out in the Red cluster with relatively low social protection expenditure per capita and was one of only two countries (alongside Czechia) rated as efficient in both DEA and NPE. Well-designed social policies may lead to high efficiency even with lower absolute public spending levels. Malta shows significant potential for enhancement. For example, the percentage of households making ends meet should increase from 11.8 to 17.9%, and Cantril scores require a substantial improvement.

In the Yellow group, Greece is an example of inefficient system. Its Cantril score should increase from 5.93 to 7.12. At the same time, the share of economically secure households must grow from 2.2 to 7.0%. Similarly, Bulgaria faces a significant challenge in the Red group: raising the economic security indicator from 2.4 to 12.6%, as recommended by NPE analysis.

Across all groups, benchmark countries usually share geographic or cultural similarity with those they influence. Finland is the model for all Scandinavian countries. Romania serves as a reference for Greece and Croatia. Estonia is a benchmark for most Eastern European countries. Regarding the guidelines for cross-regional benchmarking, countries seeking to improve efficiency should prioritise benchmarking against structurally similar countries rather than using generalised models. Policy recommendations include suggestions for realistic peer selection, i.e., inefficient countries should benchmark against neighbouring or culturally similar efficient countries. The general rule is to use incremental targeting. Policymakers should focus on achievable improvements based on empirically observed values rather than theoretical maximums.

Our analysis identified significant methodological limitations that require explicit confirmation. The DEA method sometimes produces unrealistic targets. Some performance targets exceed empirically possible values, especially concerning the Cantril ladder scores. For example, Luxembourg’s expected Cantril score exceeds the maximum possible value of 10. Similarly, Spain’s efficiency target assumes a perfect Cantril score for the entire population, which is statistically infeasible. These cases highlight a known limitation of DEA: constructing efficiency frontiers based on extreme observations. The NPE method proves superior to traditional DEA because it reduces efficiency redundancy and provides more conservative, realistic efficiency estimates. While DEA identified 6 out of 12 countries as perfectly efficient in the Eastern Europe cluster, NPE identified only 2, offering greater discrimination in efficiency levels.

## 6 Discussion

The novelty of the approach lies in the attempt to identify the types and directions of governmental expenditure on the subjective perception of well-being that generate the most significant increases. The classification approach aimed at distinguishing homogeneous subgroups among EU members preceded the implementation of the DEA (Data Envelopment Analysis) and NPE (Non-Parametric Efficiency) methods.

Analyses performed in groups showed that the optimal types and directions of expenditures are not identical for individual countries but are similar for the identified groups of countries. Such a result supports the recommendation that social policy strategies should be tailored based on identified, group-specific objectives. Group benchmarks for non-efficient countries are geographically bordering countries or those with a similar socio-political or historical-geographic background. It is an interesting observation, expected from a common-sense point of view, corroborated in both analysis variants (NPE and DEA).

Additionally, as expected, countries with highly developed social policies, i.e., Denmark, Luxembourg, the Netherlands, Austria, Finland, and Sweden, which belong to the same cluster identified due to the level of input variables, are in the same group built on output variables.

An essential result of the analyses is the observation that in some countries, the level of the output variables values, i.e., households *making ends meet* and *mean Cantril ladder score,* leads to significant differences in measurement results and policy recommendations; it applies to Spain and the Netherlands, among others. The differences indicate a direction for further research to identify the causes and formulate recommendations for the state’s social policy refining national policies. By integrating the analysis of healthcare expenditure and well-being measures, our study offers more profound insight into the quality of social policies aimed at raising well-being and promoting health improvements in EU countries.

From a methodological point of view, it is worth emphasising that the NPE method indicates a smaller number of countries considered efficient than the traditional DEA. Such a result is desirable because of the greater diversification of the indications of the input efficiency level, thus giving a better picture of the situation. Additionally, it is worth stressing that both techniques provide almost identical orders of the level of input efficiency, although there are individual exceptions, e.g., Spain (Table 1).

Applying DEA and NPE methods in analysing subjective well-being involves several significant methodological limitations. A key challenge stems from the requirement that all input variables positively correlate with all output variables. This constraint often leads to the necessity of removing variables initially identified as potential inputs or outputs. Analysing correlations between inputs and outputs is a form of preliminary efficiency screening. A negative correlation between an input and an output typically indicates that increasing the input results in a decrease in the output, which implies inefficient resource use in producing the desired effect.

The tertiary education variable did not meet the correlation requirement in this context. Higher levels of tertiary education tended to coincide with lower reported life satisfaction. Several factors might explain this outcome. Limited professional opportunities after graduation may reduce the expected benefits of higher education. Alternatively, individuals with tertiary education may interpret socio-political realities more critically, leading to lower satisfaction levels when their views conflict with prevailing policies.

DEA and NPE also pose limitations regarding how efficiency is defined. DEA measures efficiency compared to other units, while NPE considers the position within the overall system. Creating clusters of countries restricts cross-group comparisons. Despite this, observations show that units identified as efficient in the entire dataset generally maintain that status within specific groups. As a result, efficiency assessments within such groups retain broader analytical value.

Clustering countries based on similar input and output profiles improves interpretability and enables more realistic efficiency improvement goals for lower-performing units. The question “How to solve problems and become more efficient?” naturally leads to a comparative inquiry: “How do others facing similar challenges achieve higher efficiency?” Given the focus on economic determinants of subjective well-being, cultural dimensions remain beyond the scope of this analysis. For insights into the socio-cultural context of SWB, see (39).

Our findings contribute to the results of empirical research on the efficiency of public social expenditures with outcomes in subjective well-being using non-parametric methods such as DEA. Our results are consistent with recent findings of F. Sarracino and K. O’Connor (17), where the authors found that substantial expenditures on social policy do not always generate higher subjective well-being. Our results confirmed that countries with comparable social policy spendings achieve different efficiency rankings, implicating possible spending misalignment rather than insufficient investments. Our analysis also confirms M. Antonelli and V. De Bonis findings (11), which proved that countries with efficient welfare systems achieve higher life satisfaction per unit of public spending. In our study, Finland and Austria emerge as particularly notable benchmarks. Greece or Bulgaria remain inefficient even at moderate expenditure levels. The integration of DEA/NPE with the Cantril ladder and household financial security (E001) supports the OECD’s guidelines (4, 5) to include in the policy evaluation indicators beyond GDP and involve multidimensional well-being characteristics. These arguments underpin the relevance of efficiency-based approaches for assessing social investment strategies.

One of the possible directions of future research is to include some level of uncertainty in the analysis, for example, using bootstrap techniques for DEA and NPE models. Also, recently, econometric analysis of the results of the DEA model was used for the efficiency analysis of health systems in Europe (51, 52), which may be a good extension of the research described in the work. Another possible complement of the research would be performing DEA and NPE analysis using World Happiness Report indicators of happiness instead of economic measures, and also a combination of them. But such a combination of economic and WHR indicators has to be chosen carefully, because the greater the total number of variables is, the less adequate the results of DEA are.

For further research, an important issue is whether, identified in our study, marginal and partial associations represent genuine causal relationships or spurious correlations. Consistent with Simpson’s paradox, the natural way of thinking is that partial associations are actual relations, and marginal is spurious. This intuitive conclusion requires testing with empirical data. In the future, extensive investigations should focus on precisely understanding the mechanisms and role of social policy expenditures in creating better healthcare accessibility and socio-economic stability, thus generating the highest possible well-being perception.

## Data Availability

Publicly available datasets were analysed in this study. This data can be found at: https://ec.europa.eu/eurostat/web/main/data/database.
